# Implantable cardioverter defibrillator deactivation and advance care planning: a focus group study

**DOI:** 10.1136/heartjnl-2019-315721

**Published:** 2019-09-19

**Authors:** Rik Stoevelaar, Arianne Brinkman-Stoppelenburg, Anne Geert van Driel, Rozemarijn L van Bruchem-Visser, Dominic AMJ Theuns, Rohit E Bhagwandien, Agnes Van der Heide, Judith AC Rietjens

**Affiliations:** 1 Public Health, Erasmus Medical Center, Rotterdam, The Netherlands; 2 Cardiology, Albert Schweitzer Ziekenhuis, Dordrecht, The Netherlands; 3 Rotterdam University of Applied Sciences, Rotterdam, The Netherlands; 4 Internal Medicine, Erasmus Medical Center, Rotterdam, The Netherlands; 5 Cardiology, Erasmus Medical Center, Rotterdam, The Netherlands

**Keywords:** defibrillators, implantable, decision making, withholding treatment, terminal care, advance care planning, focus groups, qualitative research

## Abstract

**Objective:**

Implantable cardioverter defibrillators can treat life-threatening arrhythmias, but may negatively influence the last phase of life if not deactivated. Advance care planning conversations can prepare patients for future decision-making about implantable cardioverter defibrillator deactivation. This study aimed at gaining insight in the experiences of patients with advance care planning conversations about implantable cardioverter defibrillator deactivation.

**Methods:**

In this qualitative study, we held five focus groups with 41 patients in total. Focus groups were audio-recorded and transcribed. Transcripts were analysed thematically, using the constant comparative method, whereby themes emerging from the data are compared with previously emerged themes.

**Results:**

Most patients could imagine deciding to have their implantable cardioverter defibrillator deactivated, for instance because the benefits of an active device no longer outweigh the harm of unwanted shocks, when having another life-limiting illness, or when relatives would think this would be in their best interest. Some patients expressed a need for advance care planning conversations with a healthcare professional about deactivation, but few had had these. Others did not, saying they solely focused on living. Some patients were hesitant to record their preferences about deactivation in advance care directives, because they were unsure whether their current preferences would reflect future preferences.

**Conclusions:**

Although patients expressed a need for more information, advance care planning conversations about implantable cardioverter defibrillator deactivation seemed to be uncommon. Deactivation should be more frequently addressed by healthcare professionals, tailored to the disease stage of the patient and readiness to discuss this topic.

**Video Abstract:** 

## Introduction

The implantable cardioverter defibrillator (ICD) treats potentially lethal arrhythmias by either antitachycardia pacing or delivering an electrical shock.^1^ About half of ICD patients experience potentially painful shocks during their life.^2^ The number of ICD patients has grown, as the indication for implantation has been extended from secondary to primary prevention.^3^ While the ICD is effective in treating arrhythmias, patients eventually die due to deterioration of their underlying heart disease or another illness.^4^ An active ICD delivers shocks in the last 24 hours of life in up to 33% of patients dying non-suddenly.^5^ Shocks are potentially painful and a source of distress and anxiety for patients and relatives.^5 6^ Shocks can be avoided by timely deactivating the shock function of the ICD.^7^

International expert consensus statements from the European Heart Rhythm Association and Heart Rhythm Society recommend to timely and repeatedly discuss ICD deactivation with the patient and relatives.^1 8^ This is in line with the international advance care planning (ACP) consensus statement.^9^ ACP enables individuals to define and discuss goals and preferences for future medical treatment and care, and to record and review these if appropriate.^9^ Previous studies have shown that only a minority of patients (27% in a study from 2004,^10^ up to 35% in a study from 2018^2^) had discussed ICD deactivation with their healthcare professional and had their ICD deactivated prior to death.^2 11^ It is unknown why ICD deactivation is infrequently discussed, and what patients would want with their device when approaching the end-of-life. It is known that many patients are confused about the role of the ICD, especially in the last stages of life.^12 13^

Insight into the experiences of ICD patients with ACP conversations about ICD deactivation is limited. Having more insight into these experiences and on how ICD patients reflect on the end-of-life might help to increase the understanding of how patients think, and how they could best be approached in having an ACP conversation. Therefore, we examined ICD patients’ experiences with ACP conversations about ICD deactivation in a qualitative focus group study.

## Methods

### Study design and sample

We conducted focus groups with ICD patients, recruited via the Dutch national ICD recipient association (‘STIN’). A call for participants was published in their magazine, on their website and on social media channels. Patients were eligible when they had an active ICD, were older than 40 years, able to speak and understand Dutch and to provide written consent. Patients who were interested in participating in the study received information via email, accompanied by an informed consent form. Focus groups were organised in the Erasmus University Medical Center Rotterdam, the Netherlands. Patients were compensated for their time with a gift card of €25, and received reimbursement for travel expenses.

Forty-one patients agreed to participate in the study. Five focus groups were organised with respectively nine, eight, nine, nine and six patients.

### Data collection

Focus groups discussions were conducted in September 2017, and were led by senior researchers with experience in leading focus groups (AB-S, AVdH, JR), and supported by two researchers (RS or AB-S) who took field notes. Participants completed a questionnaire on demographic characteristics prior to the focus group. Focus groups were recorded and subsequently transcribed. Patients received a summary of the transcripts after the focus groups were conducted.

An interview guide was used to guide the focus groups (box 1). This semi-structured guide was developed by the research group, based on expert opinion and previously published literature.^13 14^ Topics discussed were: (1) information provision and communication about the ICD at the end-of-life, (2) attitudes towards ICD deactivation and (3) suggestions for improvement of information provision and communication.

Box 1 **Interview guide used to facilitate focus groups**

- Information provision and communication about the ICD at the end-of-life:
  - Did you ever had a conversation with your healthcare professional about what to do with your ICD when you get older or sick? What was discussed?
  - How do you value the quality of this conversation?
  - How would you prefer to get informed about the ICD at the end-of-life?
- Attitudes towards ICD deactivation
  - Did you ever think about what to do with your ICD when you get older or sick?
  - Would you deactivate the device? Why (not)?
- Points for improvement
  - Looking back at the conversation you had with your healthcare professional, are there things you would like to see improved?

ICD, implantable cardioverter defibrillator

### Data analysis

Transcript were analysed thematically, using the constant comparative method, a data-analytic process whereby each interpretation and finding emerging from the data is compared with previous findings.^15^ Transcripts were read by JR and RS and meaningful themes were inductively identified and summarised in a preliminary coding tree. The coding tree was discussed with the coauthors, tested on one of the transcripts, refined and finalised. Subsequently, all transcripts were coded by RS, and checked by JR. The two researchers met frequently to discuss the coded transcripts, and to discuss and resolve minor disagreements.

### Patient and public involvement

A patient advisory group met frequently for the duration of the study and provided feedback on informational materials and interview guides. At the end of the study, they commented on the findings.

## Results

Focus groups lasted an average of 97 min (range 89–107). Patients were more often male (56%) and had a mean age of 64 (SD 9.7). A majority had their ICD implanted for primary prevention (59%), and 44% had a cardiac resynchronisation therapy-defibrillator implanted, on average 6.4 (SD 4.8) years before participation in the study (table 1).

**Table 1:** Characteristics of patients enrolled in the focus groups (n=41)

Gender (male)	23 (56%)
Mean age (SD)	64.3 (9.7)
Marital status	
Unmarried	2 (5%)
Married	30 (73%)
Divorced	6 (15%)
Widowed	3 (7%)
Education	
Less than high school	1 (2%)
High school graduate	10 (24%)
Some college	8 (20%)
College graduate	15 (37%)
University degree	7 (17%)
Indication for ICD (primary prevention)	24 (59%)
Type of ICD	
Single chamber	9 (22%)
Dual chamber	6 (15%)
CRT-D	18 (44%)
S-ICD	1 (2%)
Unknown	2 (5%)
Mean years implant (SD)	6.4 (4.8)

CRT-D, cardiac resynchronisation therapy-defibrillator; ICD, implantable cardioverter defibrillator; S-ICD, subcutaneous-ICD.

The results are described along the line of the three key elements of ACP: (1) reflection on wishes and preferences; (2) discussing preferences with healthcare professionals and relatives; (3) recording and reviewing preferences.^9^ Illustrative quotes per key element are presented in table 2.

**Table 2:** Illustrative quotes regarding ACP

Elements of ACP	# Quote	Quote
1) Reflection on wishes and preferences regarding future ICD deactivation	Q1	*“I thought they implanted the ICD so I was not able to die”?* Male patient, focus group 5
	Q2	*“When do I have to do this (deactivate—RS)? If I have problems with my heart? Should I do it if I get some sort of cancer? Or suffer from dementia? When to deactivate the ICD, that is a difficult question”.* Male patient, focus group 4
	Q3	*“I have that fear (of shocks—RS) as well, and based on that fear I would say deactivate my ICD as quickly as possible when something happens. If I get sick or whatever, done with it”.* Male patient, focus group 2
	Q4	*“Well I have the ICD simply to ensure that it intervenes if(…)a cardiac arrest would occur.(…)and there is no other way (to terminate the cardiac arrest—RS) than by getting a shock. So I think I’ll welcome it then”.* Male patient, focus group 2
	Q5	*“There is of course a difference when you are terminal due to cancer(…). Because if you are terminal due to your heart disease, then such device is much more important. If you would deactivate then, then you will certainly go (die—RS). And with cancer, it can be very different”.* Female patient, focus group 5
	Q6	*“I do not want my wife, children and grandchildren to stand beside my bed, while I am bouncing up and down in my last moment. I do not want that(…)so for me, it is clear what I want”.* Male patient, focus group 4
2) Discussing preferences	Q7	*“Then he (the cardiologist—RS) told me(…)we could also deactivate halfway through. Well, I thought that was a wonderful thought”.* Male patient, focus group 3
	Q8	*“You come in as a patient, you feel like you are giving your life to such a doctor, because that is what it comes down to(…)and he does not take sufficient time to explain it properly, such an important issue as the end-of-life. And that always annoys me(…)they just do not take the time for it. You are there for ten minutes and that’s it”.* Male patient, focus group 4
	Q9	*“(…)I think the cardiologist should pay more attention to that (the ICD at the end-of-life—RS), they should realize that it is a very important topic to us”.* Male patients, focus group 4
	Q10	*“I did not have this conversation about when to turn it off, but I do not miss it at all. Because then I think well, I really am in a phase of getting better and cure, so then I think yes, I am not waiting for this (conversation about ICD deactivation—RS) at all”.* Female patient, focus group 2
	Q11	*“I have talked about it with my children, but they really do not want to know about it yet”. Female patients, focus group 5*
3) Recording and reviewing preferences	Q12	*“I do think about it (deactivation—RS) every now and then, but to record my preferences, let me just say it, that is going too far, because you do not know what it is like when you are at that moment, that you are what you recorded”.* Female patient, focus group 3
	Q13	*“I will make an advance care directive, not so much because of own situation, but because of the fact that my father died six months ago and he had one. Back then I though well, everyone should have such a document because… well, for me it felt good and I will make one myself as well”.* Female patient, focus group 2

ACP, advance care planning; ICD, implantable cardioverter defibrillator.

### Reflection on wishes and preferences regarding future ICD deactivation

Some patients were not aware that ICD deactivation was an option, and one patient thought he was not able to die with an ICD (Q1 in table 2). Of those who were aware of ICD deactivation, wishes and preferences differed. Most could imagine deciding to have their ICD deactivated one day. However, some patients indicated they could not imagine ever asking for deactivation. One patient for instance indicated that deactivating the ICD could feel like ‘euthanasia’. Euthanasia, defined as ending a patient’s life by administering medication by a physician with the explicit intention of hastening death, at the explicit request of the patient, who suffers unbearably without prospect on relief, is legalised in the Netherlands under strict criteria stipulated by the Dutch law.^16^ Some patients were uncertain about in what situation deactivating their ICD would be appropriate, stating that the decision to deactivate is complex and dependent on multiple factors (Q2).

Patients often considered the balance between quality and length of life when reflecting on possible future ICD deactivation. This balance differed between people, but generally patients indicated that postponing death—just because it is possible—without taking quality of life into account does not make sense. The ICD was considered a lifesaving device by many patients, which was also apparent in how patients talked about their device. They often used words such as ‘angel’, ‘guard dog’ and ‘safety net’. However, some patients described a ‘love-hate’ relationship with their device: previous shocks had saved them from a sudden death, but were painful, often a source of concern, and a personal confrontation with being ill and the finiteness of life. The possibility of experiencing unwanted shocks while dying was often brought up as an important factor when reflecting on the role of the ICD at the end-of-life. One patient described that their fear of receiving shocks was greater than the fear of dying (Q3). However, another patient indicated to always want arrhythmias to be treated by shocks, even if death was imminent (Q4).

Some patients stated that living longer with a compromised quality of life was undesirable. Losing independence and being diagnosed with a life-limiting illness were among the most frequently mentioned factors threatening quality of life. However, there was a distinction made in the nature of the disease. Advanced cancer or advanced lung disease was brought up by several patients as a clear indication to ICD deactivation, since an active ICD would potentially mean having to live longer with symptoms such as pain and discomfort, but, as one patient described, a progression of heart disease could make one hesitant about ICD deactivation (Q5).

Patients indicated that their relatives would play an important role in the decision-making about ICD deactivation. Several patients indicated that they did not want their relatives to witness them while receiving shocks at the end-of-life (Q6). On the other hand, some others mentioned that if their relatives would want them to continue ICD therapy, they would.

### Discussing preferences

Although patients used different sources of information, including the internet and patient folders from the hospital, the preferred mode to receive information on ICD deactivation was by having conversations with the healthcare professional. Some patients had this conversation, mostly with their general practitioner or cardiologist and on their own initiative. Such conversations gave relief to some patients (Q7). The vast majority of patients however, indicated they never had such conversations, which was source of discontent among some patients (Q8).

Patients described their cardiologist as very knowledgeable on a medical-technological level, but some indicated they felt there was not always the opportunity to discuss issues with a strong emotional component. Various reasons were suggested for this, such as a perceived lack of time or willingness of the cardiologist, as well as unawareness of importance of the healthcare professionals (Q9). These patients envisioned a greater role for other healthcare professionals to support the cardiologist in conducting these conversations, such as nurses or the general practitioner.

There was debate on the timing of conversations about ICD deactivation. Some patients thought it was best to engage early in ACP conversations about ICD deactivation, starting before implantation. However, others felt that bringing up issues related to the end-of-life at this stage might be inappropriate, since implantation of the ICD felt like a ‘second chance at life’. Therefore, some suggested to start up such ACP conversations during follow-up visits. However, others disagreed, since patients might get worried about why the physician would bring up the topic at that particular moment. Other moments were also identified to discuss ICD deactivation: when faced with a life-limiting illness, or when a do-not-resuscitate order is being discussed.

While most patients had positive attitudes towards discussing the end-of-life and ICD deactivation, this was not true for all patients. Some described they focused on living and getting better, and that they felt no need to think about future ICD deactivation (Q10).

Several patients mentioned that they had discussed possible future ICD deactivation with their relatives. Some considered such conversations to be more important than conversations with healthcare professionals. Most patients had positive experiences with talking with their relatives about ICD deactivation. Yet, some patients indicated that their relatives did not want to engage in such conversations (Q11).

### Recording and reviewing preferences

Some patients had their preferences for future medical care and treatments recorded in an advance care directive, or were planning on doing so. Not everyone was in favour of recording their preferences about ICD deactivation in a document, because they were not sure what they would want when their disease would progress. It was also described that, even if someone has well-considered ideas and wishes about what to do in specific situations, these ideas could be opposite of how someone acts when they are actually in that situation, and that people adapt to their current situation (Q12). They said that multiple factors influence the decision to deactivate, such as prognosis, age and severity of illness. Also, they were doubtful whether their wishes would be respected. On the contrary, others were in favour of reporting their preferences about ICD deactivation in an advance care directive. One patient had an earlier experience with a loved one at the end-of-life, and indicated that an advance care directive gave some clarity (Q13).

## Discussion

Little research has been conducted on the experiences of ICD patients with ACP conversations about ICD deactivation. Although international expert consensus statements recommend to timely and frequently discuss ICD deactivation with the patient,^1^ recent studies show these conversations are scarce.^2^ Several patients in our study indicated that their healthcare providers are capable regarding medical-technological issues, but felt there was not always the opportunity to discuss topics with an emotional component, such as the end-of-life. This was also shown in an American focus group study with nurses, where nurses stated that physicians sometimes ‘fail’ in considering psychosocial, economic and ACP aspects of living with an ICD.^14^ Another American interview study with ICD patients showed that patients seemed to deprioritise ACP conversations, and overemphasise the life-saving abilities of the ICD.^17^ In our study, we saw that some patients were unaware of the option of ICD deactivation. This lack of knowledge in the patient could decrease the willingness of ICD patients to engage in ACP and discuss deactivation.^18 19^ A lack of ACP conversations might impair the decision-making at the end-of-life, leading to reactive decision-making concerning ICD deactivation.^14^ Several patients indicated that it is important to discuss ICD deactivation with their relatives. However, involving families could also cause conflicts, such as relatives wanting the patient to keep the ICD active.^14^ This could be due to a knowledge deficit in the relatives as well, and could be reduced by including them in ACP conversations.^14^ The concern of family conflicts was not mentioned in our focus group discussions, although one patient mentioned to keep the ICD on if her family would want so.

There was debate on the timing of discussing ICD deactivation. Some patients were hesitant to discuss deactivation before implantation, although this would make it easier to start conversations when deactivation becomes more directly relevant.^19^ Previous research showed that conversations about ICD deactivation often only occurred when indicated during follow-up or at the end-of-life.^11 20^ Postponing the discussion until the end-of-life is not recommended, since patients might have too little time to reflect on their decision, and the last phase of life is hard to identify in patients with heart failure,^21^ as shown in a previous study, in which only 15.7% of included healthcare professionals were confident in predicting death, which might impair the timing of ACP conversations.^22^

Previous research showed that ICD patients are more reserved than other patient groups towards ACP conversations, among others because the ICD is often implanted before patients perceive themselves as being seriously ill.^13^ Also, the ICD is often advocated as a solely life-saving device.^23^ Also in our study, some patients developed a complex psychological relationship with their ICD, viewing it as a ‘trusted friend’ and an integral part of their body, which might make it difficult for patients to talk or even think about deactivating the ICD.^13 24^ In our study, some patients indicated they do not want a conversation about ICD deactivation. Although this should be respected to some extent, since ACP conversations should be tailored to the readiness and the phase of life of the patients,^9^ we do feel that the healthcare professional also has an informative role, in which all benefits, harms and future perspectives of a treatment should be discussed. Also, patients should have the opportunity to elicit general treatment preferences and goals of care in addition to their deactivation preferences, since these might play a role in their later decisions about ICD deactivation.^25^

Possibly helpful for patients might be to record their wishes and preferences in an advance care directive. In our study, only few patients did so. This was also apparent in other studies that showed that patients do often have preferences recorded on for instance feeding tubes or respirators, but are hesitant to report preferences on ICD deactivation.^26 27^ Possible explanations for this might be that preferences are subject to change, and patients are not certain whether their current wishes would represent their wishes at the end-of-life. However, recording preferences might help patients to actively think about their preferences, and could reduce ethical dilemmas or moral distress in relatives or healthcare professionals.^28^ Also, if patients are informed that the advance care directive is a ‘living’ document, which can always be adjusted, they might be less hesitant to record their wishes.

This is one of the few studies exploring the experiences of ICD patients with ACP conversations in depth. A strength is its large number of participants^29^ recruited in multiple centres. Yet, some limitations have to be addressed. Participants were recruited via the Dutch ICD patient association. It is possible that only people responded with special interest in the topic or with negative experiences, and that ACP conversations occur more often in practice. Also, it should be noted that most patients in the focus groups were in general good health, and not at the end of their lives, which might influence their wishes and preferences. Our study was conducted in the Netherlands, where there is ample attention in the public and medical professional domain to support ACP. More generally speaking, it is a country with a rather open debate about end-of-life decision-making, also showing from the issue of euthanasia that was mentioned in one of the focus groups. This means that our findings need replication in other countries and cultures.

### Implications

This study and previous studies showed a variability in how and when patients want to be informed and in their attitudes towards deactivation. In line with the international ACP consensus statement,^9^ we recommend healthcare professionals to explore the patient’s readiness to talk about end-of-life and ICD deactivation, so that information can be tailored to the needs of the patient. Specific time points to explore this are before implantation, at battery replacement, when health deteriorates or when a patient is referred to palliative care.^1^ Such conversations about ICD deactivation could be incorporated in more general conversations about goals of care, values and preferences, so that a clear understanding of the patient’s wishes could be established. We recommend to involve relatives in these conversations as well,^9^ as also indicated by the patients in our study.

## Conclusion

Many patients reflected on the role of their ICD at the end-of-life and report a need to be better informed about this topic. However, ACP conversations with the healthcare professional about treatment preferences and ICD deactivation seemed to be uncommon. Preferences about ICD deactivation were personal and dependent on the situation. Therefore, caution is advised in using one-size-fits-all approaches in informing the patient about deactivation. Some patients were hesitant to record their preferences in an advance care directive, since they were unsure whether their current preferences would reflect their future preferences.

**Key messages**

**What is already known on this subject?**

- Implantable cardioverter defibrillators (ICD) shocks at the end-of-life can be painful and a source of distress for patients and relatives.
- Advance care planning (ACP) conversations about the ICD can help patients to make a well-informed decision about future ICD deactivation, although research shows that these conversations do not occur frequently.

**What might this study add?**

- Several patients could imagine asking for ICD deactivation.
- In formulating their preferences and wishes, patients often take the balance between quality and length of life, and family preferences in consideration.
- While ACP conversations about ICD deactivation with healthcare professionals are scarce, several patients indicate they would want to engage in such discussions. Others say not to want this conversation, indicating that they focus on living and getting better.
- Some patients are reluctant to record their preferences about ICD deactivation in an advance care directive.
- Patients indicate that their preferences are dependent on the situation, and they are unsure whether current preferences would reflect future preferences.

**How might this impact on clinical practice?**

- Although many patients want to be informed about ICD deactivation, there is no one-size-fits-all approach for ACP, since there is variability in how and when patients want to be informed, and in their attitudes towards deactivation of the ICD.
- Healthcare professionals should address the role of the ICD in the last phase of life more often, tailored to the readiness of the patients to engage in these conversations and the phase of life the patient finds himself/herself in.
- The balance between quality and quantity of life should more often be discussed with the patient in clinical practice.
